# Improving Pacific Adolescents’ Physical Activity Toward International Recommendations: Exploratory Study of a Digital Education App Coupled With Activity Trackers

**DOI:** 10.2196/14854

**Published:** 2019-12-11

**Authors:** Olivier Galy, Kalina Yacef, Corinne Caillaud

**Affiliations:** 1 Interdisciplinary Laboratory for Research in Education, EA 7483 School of Education The University of New Caledonia Noumea New Caledonia; 2 School of Computer Science The University of Sydney Sydney Australia; 3 Charles Perkins Centre The University of Sydney Sydney Australia; 4 Faculty of Health Sciences The University of Sydney Sydney Australia

**Keywords:** exercise, eHealth, adolescents, health education, noncommunicable diseases, iEngage, data mining, movement, food, Melanesia

## Abstract

**Background:**

The prevalence of overweight and obesity in children and adolescents has dramatically increased in the Pacific Island countries and territories over the last decade. Childhood overweight and obesity not only have short-term consequences but are also likely to lead to noncommunicable diseases in adulthood. A major factor contributing to the rising prevalence is an insufficient amount of daily moderate-to-vigorous physical activity (MVPA). In the Pacific region, less than 50% of children and adolescents meet the international recommendations of 11,000 steps and 60 min of MVPA per day. Although studies have shown the potential of digital technologies to change behaviors, none has been proposed to guide adolescents toward achieving these recommendations.

**Objective:**

The aims of this study were (1) to investigate whether a technology-based educational program that combines education, objective measures of physical activity (PA), and self-assessment of goal achievement would be well received by Pacific adolescents and help change their PA behaviors toward the international PA recommendations and (2) to create more insightful data analysis methods to better understand PA behavior change.

**Methods:**

A total of 24 adolescents, aged 12 to 14 years, participated in a 4-week program comprising 8 1-hour modules designed to develop health literacy and physical skills. This self-paced user-centered program was delivered via an app and provided health-related learning content as well as goal setting and self-assessment tasks. PA performed during the 4-week program was captured by an activity tracker to support learning and help the adolescents self-assess their achievements against personal goals. The data were analyzed using a consistency rate and daily behavior clustering to reveal any PA changes, particularly regarding adherence to international recommendations.

**Results:**

The consistency rate of daily steps revealed that the adolescents reached 11,000 steps per day 48% (approximately 3.4 days per week) of the time in the first week of the program, and this peaked at 59% (approximately 4.1 days per week) toward the end of the program. PA data showed an overall increase during the program, particularly in the less active adolescents, who increased their daily steps by 15% and ultimately reached 11,000 steps more frequently. The consistency of daily behavior clustering showed a 27% increase in adherence to international recommendations in the least active adolescents.

**Conclusions:**

Technology-supported educational programs that include self-monitored PA via activity trackers can be successfully delivered to adolescents in schools in remote Pacific areas. New data mining techniques enable innovative analyses of PA engagement based on the international recommendations.

## Introduction

### Background

The populations of the Pacific Island countries and territories (PICTs) have undergone a rapid lifestyle transition, with impacts on health. Despite a history of colonization, tribal and rural living has persisted, but today’s urbanization, industrialization, and mechanization are causing many transformations [1], notably a decrease in physical activity (PA) and dietary changes because of imported and processed foods [2]. These phenomena have affected the main PICT communities (Melanesian, Polynesian, Asian, and European) [3,4], resulting in an alarming increase in overweight and obesity in children and adolescents [5] that often persists into adulthood [6] and leads to noncommunicable diseases [7]. Early intervention is, therefore, crucial to reverse these trends, and both PA—especially moderate-to-vigorous physical activity (MVPA)—and healthy dietary behaviors in childhood are notably associated with a healthy adult lifestyle [3]. Research indicates that less than 50% of children aged between 13 and 15 years living in the Pacific meet the international recommendations of 11,000 steps and 60 min of MVPA per day [2]. In New Caledonia, 35% of children aged between 11 and 16 years are overweight or obese [3,4], and 45% and 20% of these adolescents living in rural and urban regions, respectively, meet the recommendations [8]. Although these data are based on self-reports, which are known to have a large margin of error when estimating PA levels, they underline the need for action to encourage children and adolescents to become more physically active [9]. This is a challenge for the PICTs and indeed many countries worldwide.

Data are limited for the PICTs, but a recent Australian study found that initiatives to increase adolescent MVPA have been unsuccessful [10]. The low MVPA was attributed in part to perceived lack of support, poor motivation, and low physical competence [11]. Important drivers for children’s and adolescents’ engagement in PA include health knowledge, personally organized PA, and competence in diverse PA types [12]. Systematic reviews of Western studies have concluded that future initiatives need to focus on school interventions involving families or communities and should embed multiple components, such as the guided use of technology [13,14].

Furthermore, digital interventions aiming to increase PA have shown promising results, particularly when they involve self-monitoring and feedback via activity trackers [15]. However, the optimal choice and appropriate use of the trackers is critical; Kerner and Goodyear [16] indicated that a bracelet and app designed for adults but used for adolescents without educational support conveyed inappropriate messages (eg, *be fit or be fat*), resulting in demotivation and negative feelings.

We explored a technology-supported educational program focused on PA in a Pacific Island rural school environment. Our program (called iEngage) was designed to improve adolescents’ PA knowledge and skills and to help them understand their activity tracker data, which provided objective feedback on their PA. Data mining enabled us to develop a comprehensive approach to analyzing PA trends throughout the program. First, clustering took into account all the components of the international recommendations (number of steps, time spent at each intensity, and days over 11,000 steps). Second, a sliding 7-day window captured the frequency and regularity of the PA behaviors.

### Objectives

The first aim was to investigate whether a technology-based educational program that combines education, objective measures of PA, and self-assessment of goal achievement would be well received by Pacific adolescents and help change their PA behaviors toward the international PA recommendations. The second aim was to create more insightful data analysis methods to better understand PA behavior change.

## Methods

### Study Design and Participants

This exploratory study was conducted in a rural school in New Caledonia on Lifou Island. A total of 24 adolescents aged 12 to 14 years participated in this pilot. Parents gave informed written consent before their child’s participation in the study, which met all legal requirements and the criteria of the Declaration of Helsinki. The protocol was approved by the ethics committee of the University of New Caledonia and the consultative ethics committee of New Caledonia (CEC-NC03-2016).

**Figure 1:**
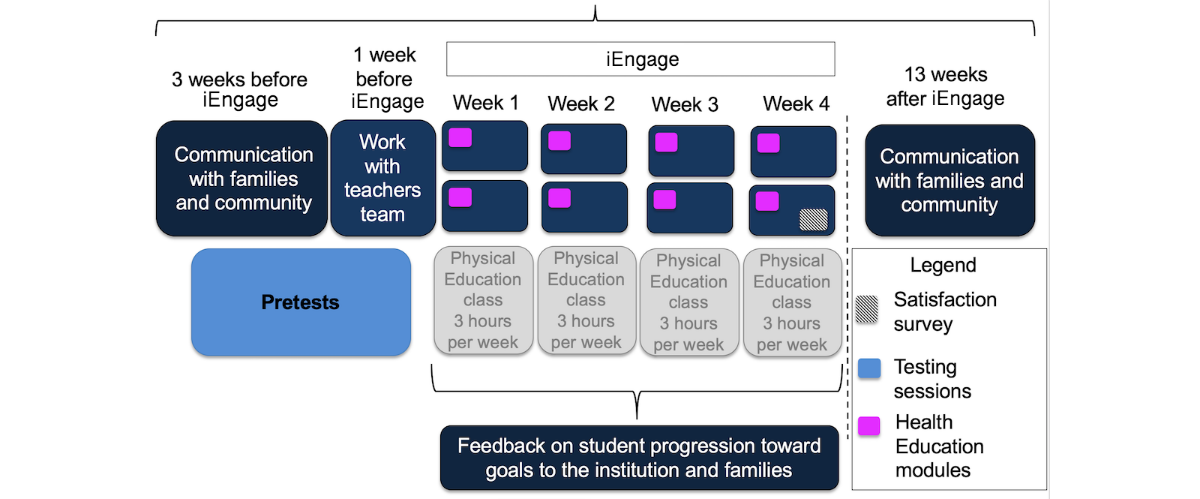
Study design. Pretests and communication with families began 3 weeks before the start of the educational program; coordination with the teaching team was finalized 1 week prior, along with baseline measures. The digital program lasted for 4 weeks, with biweekly health education modules held in class (in pink). Feedback to families about the program occurred in the week following the end of the program.

Our study design is illustrated in Figure 1. Before the study, we communicated extensively with the school principal, the teaching team, and the wider community (including families) to explain the framework of the iEngage educational program.

Moreover, 3 weeks before the program, anthropometric and physical fitness data including aerobic capacity, speed, and agility were assessed during scheduled physical education classes as described below. Once anthropometry and physical fitness testing were completed, participants were equipped with research-grade activity sensors (GENEActiv) for 5 consecutive school days before the iEngage program to measure baseline PA behavior. They then started the iEngage program, which lasted for 4 weeks, with 2 1-hour modules per week. Throughout the program, they continuously wore a commercial activity tracker (Misfit Shine 2, United States), which served as the educational support tool. The 2 activity trackers served very different purposes and collected PA data in different formats. To measure baseline PA, we needed a device meeting the research-grade criterion, and to support learning during the program, we needed a device that was commercially and easily available for future wide-scale deployment.

### iEngage: A Technology-Enabled Educational Program

**Figure 2:**
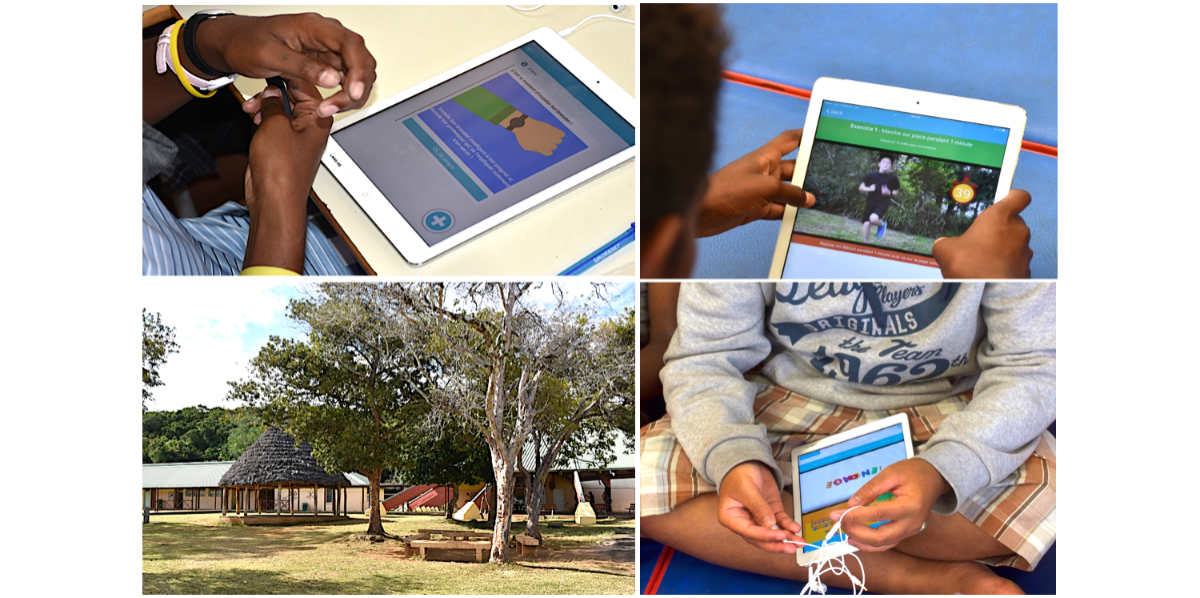
App screenshot and pictures of children in activity during modules.

The iEngage program targets health literacy, PA-related skills, and sugar-focused nutrition guidelines. Health literacy topics include the definition of PA, classifications of PA intensities (light, moderate, and vigorous), physical fitness parameters and their interpretation, rating one’s own effort during exercise, sedentary behaviors and their effects on health, health definition, well-being (physical, mental, and social), sugar as a source of energy, and guidelines related to sugar consumption. iEngage v1.0 was delivered via an app (powered by BePatient) comprising 8 1-hour modules; it was self-paced and had learning activities as well as goal setting and self-assessment tasks. Each module proposed learning activities, quizzes, and brief 2- to 5-min PA sessions that generally focused on a particular series of movements: sprints, jumping, walking, running, squats, sit-and-reach, or push-ups. The total PA per adolescent over the 8 modules was 21 min; thus, these prescribed PA sessions had no significant direct impact on the MVPA levels and could not have contributed to an artificial increase in MVPA. PA was captured by the Misfit Shine 2 activity tracker and conveyed to the child via the Misfit app exclusively during the iEngage modules to support learning and provide help in self-assessing achievement against personal goals. Figure 2 presents screenshots of the app and pictures of children in activity during the program.

At the end of each module, the adolescents were guided to set their own goals for the next module. These were 2-fold: first, they chose an individual “objective” in terms of number of steps and intended intensity (eg, “I plan to do 10,000 steps and 45 min of MVPA every day”), and then, they selected a “mission,” which was a specific task usually involving family, friends, or health professionals (eg, “I will discuss what I’ve learned today with my family” or “I will play soccer with friends”). At the start of the following module, they indicated whether they had achieved their goals. In the last module, they were encouraged to set long-term goals.

A game challenge was designed to foster engagement in learning activities and goal achievement. The adolescents were assigned to 5 groups of 3 or 4, represented by an animal mascot. Groups won points when members achieved individual goals (objectives and missions) and for collective learning achievements during the modules; the results were tallied at the end of each week.

### Procedures

#### Preprogram Measurements

##### Anthropometry

Height was measured to the nearest 0.5 cm using a portable stadiometer (Leicester Tanita HR 001, Tanita Corporation, Tokyo, Japan). Body weight was determined using a scale (Tanita HA 503, Tanita Corporation, Tokyo, Japan) to the nearest 0.1 kg, with the adolescents in light clothing. Body mass index (BMI) was calculated by dividing mass in kilograms by height squared in meters. The BMI z score was then calculated. The percentage of fat body mass (FBM) was estimated from the skinfold thickness of the sum of 4 skin areas (biceps, triceps, subscapular, and supra-iliac) measured on the right side of the body with Harpenden skinfold calipers, expressed in millimeters. This enabled us to determine the FBM and lean body mass) in kg. The detailed anthropometric methods are described in the study by Galy et al [17].

##### Physical Fitness Tests

Physical fitness tests included a time trial of 30-m sprints, with 5 m, 10 m, and 15 m lap times recorded using photocell gates (Brower Timing Systems, Salt Lake City, UT, United States; accuracy of 0.01 sec) placed 1 m above the ground. The *t* test was used to determine the adolescents’ agility. Trials were recorded using photocell gates in the same conditions as for the sprints. The maximal aerobic speed (MAS) test individually assessed running speed over gradually accelerating 1-min increments. When a child stopped the test at maximal effort, the last stage reached was recorded and converted to MAS as described in the study by Galy et al [17].

##### Baseline Physical Activity (GENEActiv)

Baseline PA was obtained via GENEActiv activity trackers (validated in children [18]; Kimbolton, Cambs, PE28 0LF) positioned on the nondominant wrist for 5 days before the program. The datasets from these trackers contained 60-Hz 3-dimensional accelerometer data. Raw data were processed into 1-sec epoch signal vector magnitude datapoints of daytime activity (8 am to 8 pm daily) and were then categorized into PA levels: sedentary, light PA, and MVPA, for each second using Phillips’s cut points for children [19]. We focused on bouts of sedentary activity or MVPA occurring with a minimum duration [18], as these behaviors were targeted by the program. A bout was defined as a continuous episode of PA at a specific range of intensity, and the length of a bout was the number of seconds spent at that intensity during that episode. Thresholds for sedentary bouts (60 sec minimum) and MVPA (3 sec minimum) were based on the literature [18] and the analysis of Diaz and Yacef [20].

##### Physical Activity During the Program (Misfit)

The Misfit activity trackers were worn continuously over the 4-week program, and data were available to the researchers in a daily aggregated form for each child: this included the total daily steps and the PA sessions that were detected with duration, steps, and calories spent during each session. We reverse-engineered the PA intensities from calories spent using the following criteria, which were found empirically to match Misfit’s categories: PA sessions that consumed less than 2 calories per minute were considered light, between 2 and 3.5 calories were considered moderate, and over 3.5 calories were considered vigorous. These empirically defined cut points are aligned with those of Colley et al [21] for light and moderate PA and were slightly lower for vigorous activity, suggesting that our calculation was on the conservative side. This had no bearing on our study as we were interested in MVPA.

As noted earlier, these trackers were not used here for scientific validation but for learning purposes. Nevertheless, to assess data accuracy, we compared their data with those of the GENEActiv trackers, by asking children to wear them concurrently on the same wrist during the first week: the daily totals were consistent, although the GENEActiv trackers sometimes captured more finely detailed data. This indicates that the Misfit trackers may sometimes underestimate very small activity bouts, yet still provided a close lower bound of the children’s activity. Their continuous data across the 28-day program data were, therefore, useful to explore.

### Feedback From Adolescents

At the end of each module, the adolescents had the opportunity to provide quick feedback on their experience via a survey question: “How much fun was this module?”

At the end of the program, through a multiple-choice quiz, they were also asked to indicate their intention to engage in healthier PA behaviors in the coming months.

### Statistical Analysis

#### Anthropometry and Physical Fitness Analysis

Anthropometry and physical fitness data are presented as mean (SD). Means and SDs for the physical fitness tests were calculated for the whole group; these tests were MAS; speed at 5, 10, 15 m; and an agility test.

#### Analysis of Baseline Physical Activity (GENEActiv)

The mean and SD of daily times spent being sedentary and engaged in light, moderate, and vigorous PA were calculated on the minimum bout-filtered daily accumulated data. Analyses were conducted using R Core Team 3.1.0. [22].

### Data Mining Methods

#### Analysis of Program Physical Activity (Misfit)

We analyzed the PA data in several ways to gain insight into the PA behavior changes during the program. We first identified the types of daily behavior observed through *daily behavioral clustering*. We then devised a *consistency rate* function to track the regularity of the participants’ healthy PA behaviors over a sliding window of 7 days. We assumed that although the adolescents might not meet the PA recommendations every day, a noteworthy sign of progress is the fact that they meet them more regularly at the end of the program than at the beginning. These methods are summarized below.

#### Daily Behavior Clustering

In addition to the binary achievement of minimum daily steps, we explored different shapes of the PA behaviors regarding the number of steps, the time spent at various intensities, and how these behaviors changed daily. The behavior clustering method thus revealed the types of PA behaviors over a day. The activity tracker data, for each child and each day, were mapped into vectors with 5 features: 1, 2, and 3—the total daily time spent in light, moderate, and vigorous PA, respectively; 4—the total number of steps; and 5—a binary feature that was true if at least 11,000 steps were accumulated on that day. Each of these vectors was, therefore, a representation of the PA behavior of 1 child on a given day. Numerical features were normalized.

These vectors of daily PA behaviors were then clustered (Kmeans, k=3 determined using the elbow method to find the best balance between the number of clusters and the sum of square errors within clusters).

#### Consistency Rate

**Figure 3:**
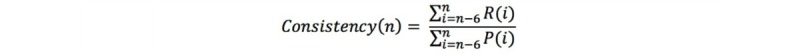
Consistency equation.

The consistency rate captures the frequency and regularity of desirable behaviors in relation to a specific target. It represents the average value of achievement of that target over a certain number of days, here 7, and is computed as shown in Figure 3, where *n* is the number of days (ie, 7), *R(i)* is the degree of target achievement on day *i*, and *P(i)* is a binary function showing whether behavioral data were available on day *i*.

The purpose of *P(i)* was to ensure that the data were only averaged for the number of days on which data were captured and to exclude those days when the adolescents did not wear their device (loss, malfunction, etc). For instance, an adolescent who had not once met the recommendations over the prior 7 continuous days would have a rate of 0%, whereas one who did so consistently would have a rate of 100%. If no data were recorded in the 7-day period, the value was excluded. As this 7-day window moved each day, we were able to follow trends with the consistency rate.

To follow progress, we applied this method to 2 targets: (1) daily number of steps over 11,000 and (2) daily PA behavior within the desirable clusters. For the first target, R(i)=1 if the daily number of steps were achieved on day i, 0 otherwise. For the second one, each cluster was assigned a value within the range (0;1) corresponding to the activity level of that cluster (where 1: most active and meeting all international recommendations), and R(i) was the value of the corresponding behavior cluster on day i.

## Results

### Anthropometry, Physical Fitness, and Physical Activity Before the iEngage Program

Descriptive data collected before the iEngage program are presented in Table 1. Accelerometry results from GENEActiv show that participants spent an average of 112 min per day in sedentary behaviors lasting at least 60 sec. Daily (8 am-8 pm) baseline PA intensities expressed in minutes (sedentary, light, and MVPA) showed that they spent 122 min in light PA and 36 min in MVPA in bouts lasting at least 3 sec.

**Table 1:** Descriptive anthropometric, physical fitness, and physical activity data before the iEngage program (N=24).

Individual characteristics		Value, mean (SD)	
**Anthropometric variables**			
	Height, m		1.58 (0.06)
	Weight, kg		56.45 (13.09)
	Age, years		11.88 (0.57)
	FBM^a^, %		27.99 (7.52)
	FBM, kg		39.99 (6.76)
	BMI^b^, %		22.33 (4.60)
	BMI z score		1.20 (1.02)
	Waist, cm		81.58 (10.84)
	Waist to height ratio		0.51 (0.06)
**Physical fitness variables**			
	Maximal aerobic speed, km.h^-1^		10.90 (1.10)
	5 m sprint, sec		1.36 (0.10)
	10 m sprint, sec		2.96 (0.33)
	30 m sprint, sec		5.38 (0.44)
	Agility test, sec		13.02 (3.09)
**Mean time spent per day at the corresponding intensity in minutes**			
	Sedentary activity (60-sec bouts)		112.20 (19.30)
	Light physical activity (3-sec bouts)		121.90 (5.80)
	Moderate-to-vigorous physical activity (3-sec bouts)		35.30 (3.80)

^a^FBM: fat body mass.

^b^BMI: body mass index.

### Daily Behavior Clustering

Behavior clustering identified 3 types of daily PA behaviors, shown in Table 2: the less active cluster (cluster 0), well under the daily recommended number of steps and minimal MVPA; the active cluster (cluster 1), where daily step count was reached but well under 60 min of MVPA; and the very active cluster (cluster 2), with active days and well over the recommended step number and time in MVPA.

**Table 2:** Centroids of daily behavior clusters (k=3) during the 4-week iEngage program.

Feature	Mean cluster 0: less active cluster (n=180)	Mean cluster 1: active cluster (n=229)	Mean cluster 2: very active cluster (n=37)	Mean overall (n=446)
Light-intensity activity, min	39	92	85	64
Moderate-intensity activity, min	5.30	16	57	14
Vigorous-intensity activity, min	2.30	4	52	7
Number of steps	7850	13,860	18,260	11,140
Over 11,000 steps	No	Yes	Yes	No

### Consistency Rate

Progress was assessed according to 2 daily targets: (1) reaching 11,000 steps and (2) being in the most active daily PA behavior cluster.

#### Target 1: Achievement of Daily Steps

**Figure 4:**
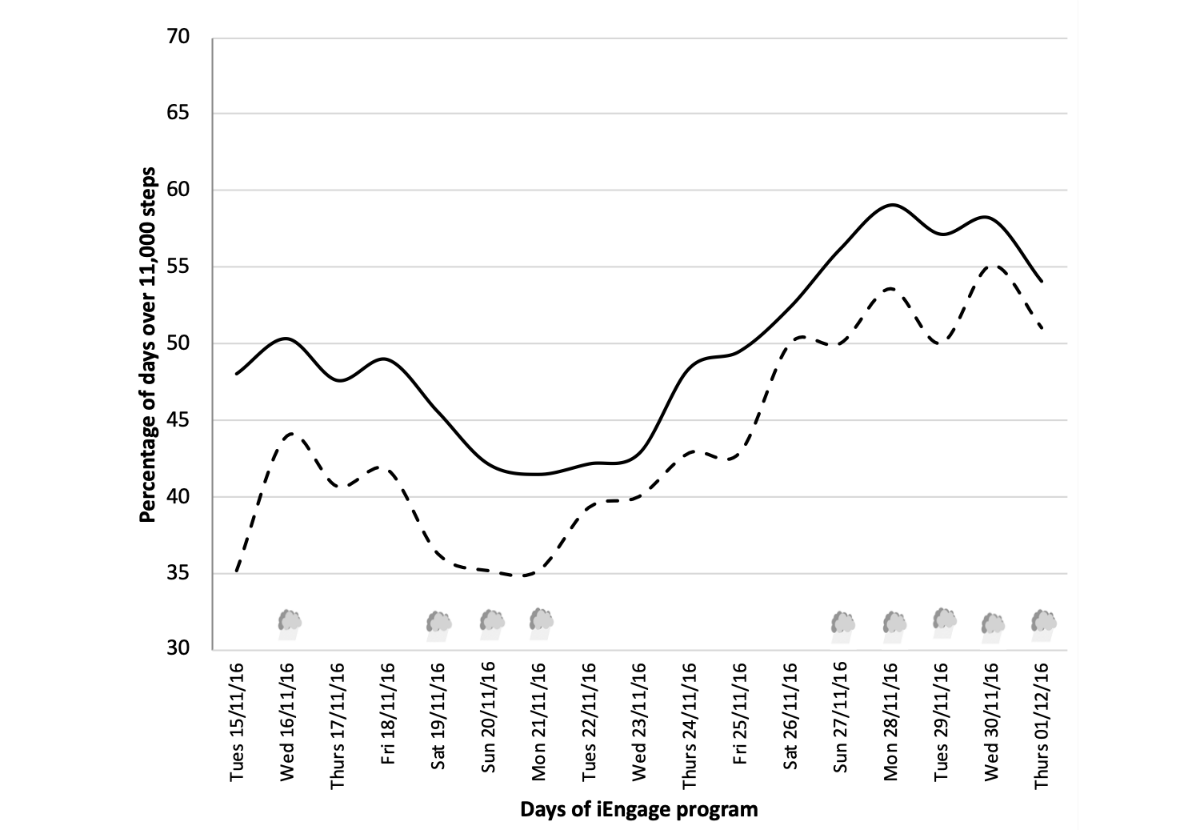
Average consistency rate (in %) of daily steps during the iEngage program. Consistency rate is calculated at the end of a 7-day window sliding over time; hence, the data presented in the graph start on day 7. Rainy days (N=10, between 0.3 and 14.3 mm.3h^-1^) are represented by a cloud symbol.

Overall analysis showed that, on average, adolescents achieved 11,197 (SD 1376) steps per day during the 4-week program. The consistency rate of achieving 11,000 steps per day improved throughout the program (Figure 4). Consistency rate analysis showed that 11,000 steps were achieved for 48% of the days in week 1 (approximately 3.4 days/week). This increased to 54% (approximately 3.8 days/week) in week 4, with a peak of 59% (approximately 4.1 days/week) shortly before. More interestingly, the adolescents who were the least active in week 1 (ie, achieving less than 50% of daily recommendations in the first week) increased their consistency rate from 35% (approximately 2.4 days/week) at program start to 51% (approximately 3.5 days per week) at program end, with a peak of 53.6% (approximately 3.7 days/week).

#### Target 2: Improvement in Daily Physical Activity Behavior

**Figure 5:**
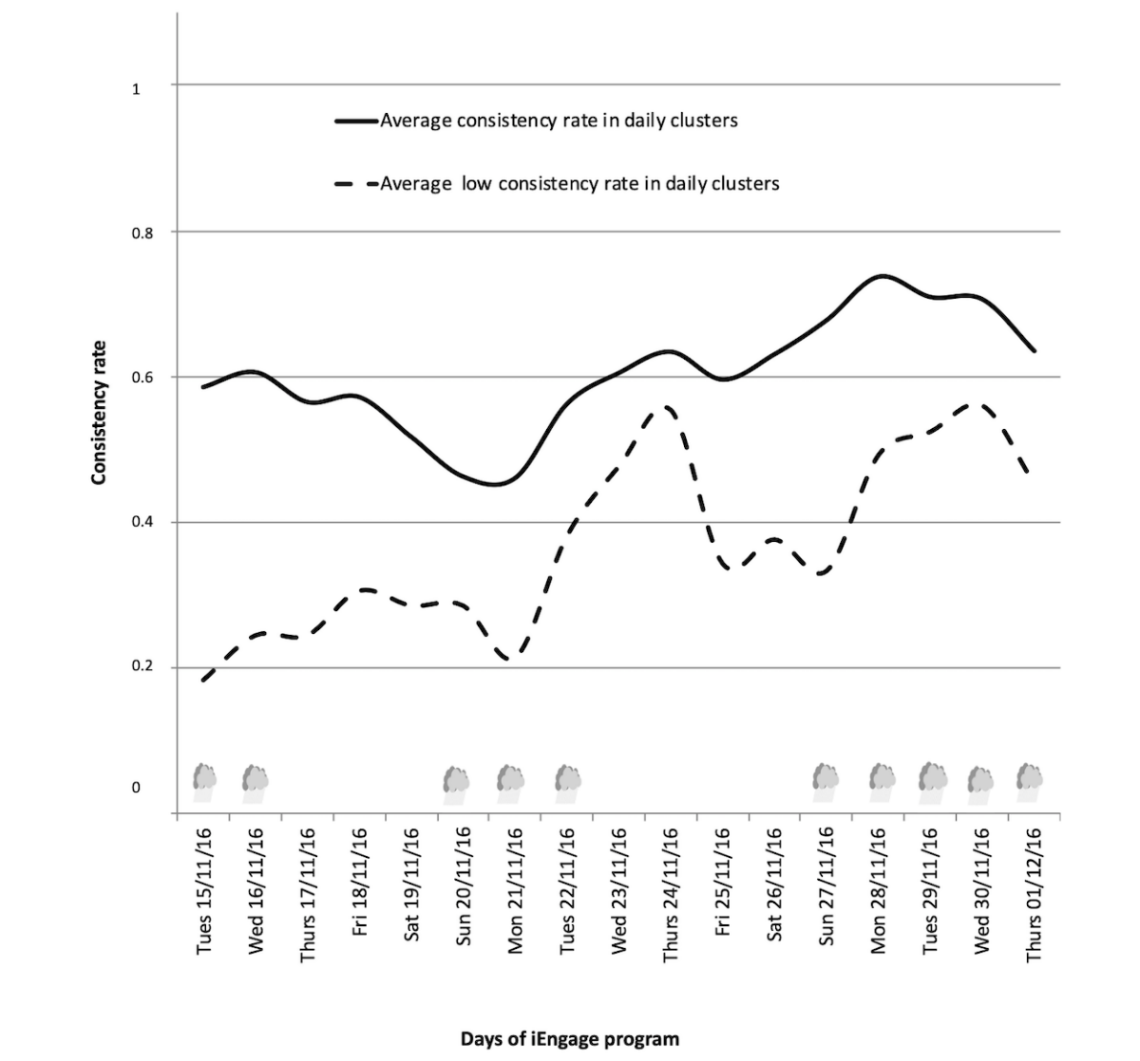
Average consistency rate (from 0 to 2) of daily behavior clusters during the iEngage program. Rainy days (N=10, between 0.3 and 14.3 mm.3h^-1^) are represented by a cloud symbol.

The daily behavior clusters provided a more refined analysis of progress, taking into account the time spent at each PA intensity (light, moderate, and vigorous), the number of steps, and the achievement of daily recommended steps. With 3 clusters, R(n) was a 3-value function: 0 for cluster 0 (less active), 0.5 for cluster 1, and 1 for cluster 2 (very active, meeting all recommendations). A consistency value of 0 meant that every day of the past week was spent in the less active cluster, and a value of 1 meant that every day was spent in the very active cluster. A value of 0.5 could mean that the adolescents split their time between cluster 0 and cluster 2 (alternating less active and very active days) or every day in cluster 1 (active but under the recommendations). The analysis of weekly consistency rate using these clusters is presented in Figure 5. On average, the adolescents started at 0.60 and finished at 0.63 (with a peak at 0.71) at the end of the iEngage program. More interestingly, the least active adolescents in week 1 (defined as being in the less active cluster more than 50% of the time) started with a consistency rate of 0.18 and ended with 0.45 (peak at 0.51), indicating a 27% increase over the program for these adolescents.

### Feedback From Adolescents

The percentage of satisfaction with the modules (ie, module rated as “fun”) was 95% across the program (for all modules). At the end of the program, adolescents were invited to set some goals for the future. Overall 85% (18/21) of adolescents declared intentions to achieve daily MVPA: 52% (11/21): “I intend to achieve 60 min of MVPA per day” and 33% (7/21) “I intend to achieve 30 min of MVPA per day”. Furthermore, 62% (13/21) declared “I will discuss with my family and friends about what I learned,” 62% (13/21) “I will seek assistance from close friends to reach my objectives,” and 0% “I don’t know/I am not motived.” To the question “Do you think you will achieve it?,” the answer was “Yes” for 38% (8/21) of the adolescents, “I’m not sure” for 57% (12/21), and “I don’t think so” for 4% (1/21).

## Discussion

This study showed that deploying a technology-supported educational program in a school context targeting PA behaviors in a remote area of the PICTs is feasible. In addition, we provide new data mining techniques for tracking adolescents’ adherence to international PA recommendations through daily behavior clustering and consistency rate.

The 95% Melanesian population was from a remote and rural area of New Caledonia. The overweight prevalence and the physical fitness level before the program (Table 1) were consistent with previous observations in the region [7,11,23-25]. PA data recorded before the program, although in a small group, showed for the first time that these adolescents performed only approximately 30 min of MVPA daily, which is half the recommended volume (Table 1). This is similar to other Asia-Pacific countries such as Australia [10] and highlights the need for intervention for New Caledonian adolescents [8].

PA was analyzed using novel integrative approaches. First, it was analyzed per day and per adolescent rather than averaged over the observed period. This aligns with how recommendations are expressed and allowed the detection of changes on a daily basis. Second, a behavior clustering technique was used to combine the PA components to characterize the daily PA of an adolescent: volume (daily steps), time spent at each PA intensity, and achievement of internationally recommended daily steps. These objectively measured data enabled us to identify 3 daily PA behavior clusters (Table 2): days spent in clusters 0 and 1 were below the international recommendations of 60 min of MVPA per day, whereas days in cluster 2 (only 37/446 days) were very active and well over the international recommendations. Third, changes in PA were measured by calculating the consistency of PA over a 7-day sliding window. PA was measured via 2 indicators: a simple one based on the achievement of the recommended daily steps (Figure 4) and a more comprehensive one using the daily behavior clusters (Figure 5). Figure 4 shows that the least active adolescents in the first week (eg, achieving the daily recommended steps less than half the time) progressively increased their steps by 15.6% over the 4-week program. Furthermore, the consistency rate and daily behavior clustering methods that take into account durations, intensities, and recommendations were useful for interpreting the PA patterns. This integrative approach showed that the adolescents improved PA behaviors over the program (+27%; Figure 5), especially those who were the least active in the first week. Indeed, these adolescents more often met the international recommendations for daily steps and PA intensity by week 4. This approach provided better daily global analyses of PA patterns in adolescents based on the recommendations [26].

Overall, PA steadily increased, showing levels approaching the international recommendations, especially for those who were less active at the beginning of the study. We highlight that PA was realized outside the iEngage modules, ie during the adolescents’ daily life, as the program only prescribed at most 4 min per module (the remaining time in each session was spent learning via the app or discussing concepts with peers).

The school educational team, including the principal, the teachers, and the school nurse, as well as the families and community (Melanesian custom) were very involved in the program, as we intended it. Indeed, a New Zealander study showed that contextualized learning facilitated knowledge translation in programs promoting scientific and health literacy [27]. The adolescents were more able to decide whether and how to incorporate scientific evidence to enhance their current and future well-being. The program encouraged them to share knowledge and skills with peers, family, and the broader community (including teachers and school nurse) and to involve them in achieving health goals. For example, as the efficacy of adolescent health interventions depends in part on family involvement [14], these adolescents were encouraged to identify a “mission” at the end of each module to engage parents, carers, siblings, or friends in the activity and/or discuss health information and recommendations. We put in place an information strategy and meetings with parents along with important people in the community, eg, religious leaders and elders. Some of them accepted to convey messages of support for the program based on their teaching discipline or role in the community. They did so in French and the local language (*Drehu*) via videos that were incorporated into the digital program. This was particularly important because of the key role of spiritual leaders in Pacific communities [28]. In particular, we invited “custom keepers” and religious leaders to talk in *Drehu* about the importance of PA and nutrition as ways to build healthy lifestyles and be part of healthy communities. We believe that this community involvement contributed to the adolescents’ strong appreciation for the program and their intentions to continue their engagement after the program end, and these too were positive outcomes of this exploratory study.

Some study limitations need to be underlined. This exploratory study was conducted in 1 class with 24 children for a period of 4 weeks and no control group was included, which limits any generalization. Although Misfit data were recorded continuously during the program, enabling us to obtain unique information on the children’s behavior, we did not measure the pre-post effects of the intervention via accelerometry, and this may have limited the interpretation. However, at the start of the program and for 5 consecutive days, the Misfit data were compared for data accuracy with the GENEActiv data. The daily totals were consistent, indicating that although the Misfit trackers sometimes underestimated small activity bouts, they still provided a close lower bound of the children’s activity.

In conclusion, an integrative approach in the sports sciences, combining user-centered and community-based education and digital technologies (wearable trackers) to build health-related skills and change PA behaviors, seems promising in the PICTs. Our novel data mining technique provides an innovative way to assess adherence to international PA recommendations. Although several improvements are needed, this exploratory study showed that an electronic health program can be deployed in a remote Pacific area with a strong traditional culture and suggests the potential for implementing it at a larger scale for different ages, populations, and cultures.

## Abbreviations

BMI: body mass index
FBM: fat body mass
MAS: maximal aerobic speed
MVPA: moderate-to-vigorous physical activity
PA: physical activity
PICTs: Pacific Island countries and territories
